# Detection of different enteric viruses in children with diarrheal disease: evidence of the high frequency of mixed infections

**DOI:** 10.1099/acmi.0.000010

**Published:** 2019-03-29

**Authors:** Vaishali S. Tatte, Varanasi Gopalkrishna

**Affiliations:** 1 Enteric Viruses Group, National Institute of Virology, Pune, India

**Keywords:** multiplex RT-PCR, enteric viruses, diarrhoea, fecal specimens

## Abstract

Enteric viruses play a major role in causing diarrhea in children. Early identification of the causative pathogen is still a challenge in the clinical laboratory. A multiplex PCR assay is a useful tool to screen a large number of clinical samples especially in an outbreak situation. In this study, a multiplex reverse transcription (RT)-PCR assay was developed to detect nine enteric viruses such as group A rotavirus, norovirus GGII, sapovirus, adenovirus, astrovirus, aichivirus, parechovirus, bocavirus and enterovirus in clinical samples of diarrheal cases. Stool samples (*n*=185) collected from infants and children with acute gastroenteritis cases in Pune, western India were analysed for nine different enteric viruses by currently developed multiplex RT- PCR. Predominance of group A rotavirus (76%) followed by enterovirus (11.5%), astrovirus (4.5%), adenovirus (2.7%) and norovirus GII (1.6%) was observed. A total of 44.8 % (82/185) samples analysed by this method showed high frequency of mixed infections. These results highlighted high prevalence and diversity of different enteric viruses in children. The multiplex PCR showed good concordance with monoplex RT-PCR for detection of these enteric viruses in clinical samples. This is the first report on the development of a multiplex RT-PCR assay for detection of multiple enteric viruses in diarrheal diseases from India.

## Introduction

Diarrhea caused due to viral and bacterial infections is a major public health problem in developing countries. It has been estimated that 4–38% of deaths among children <5 years of age are caused by viral infections [1]. Clinical presentation of the patients with diarrheal symptoms is not generally indicative of any pathogenic agent [2, 3]. Among enteric viral pathogens, group A rotaviruses (RVA) family *Reoviridae* followed by noroviruses (NoV) family *Caliciviridae*, enteric adenoviruses (AdV) family *Adenoviridae*, astroviruses (AstV) family *Astroviridae*, sapoviruses (SaV) family *Caliciviridae* have been found associated in acute gastroenteritis [4]. In recent years, several novel enteric viruses such as aichivirus (AiV) family *Picornaviridae, genus Kobuvirus*., enteroviruses (EV) family *Picornavirus,* human parechoviruses (HPeV) family *Picornavirus,* sali (SaliV) family *Picornavirus,* and human bocaviruses (BoV) f*amily Parvoviridae* have also been found associated with acute gastroenteritis [2, 5–8].

Conventional diagnostic methods for routine detection of enteric pathogens rely mainly on microscopy, virus isolation and enzyme immunoassays. However, these methods are either laborious or with limited sensitivity and specificity. For conducting epidemiological studies, use of reverse transcription (RT)-PCR and sequencing techniques have become the standard methods for detection and characterization of enteric pathogens, which includes bacterial and viral pathogens [6, 9–15]. In the past, multiplex real-time PCR has been successfully used to identify different enteric pathogens simultaneously [3, 14–17]. However, these methods are either low through-put or expensive (involvement of fluorescence probe), which does not allow rapid screening of a large number of stool samples to be analysed in an outbreak situation. Although several multiplex molecular assays such as Luminex GPP, Film Array and Seegene Diarrhea ACE kits are commercially available for testing, these along with gastrointestinal pathogen panel (GPP) tests, are extremely unaffordable methods for routine use [18, 19]. In a developing country such as India, where diarrhea remains as a leading cause of death, identification of the most predominant circulating viral etiological agents will be useful in development of intervention strategies to control disease. A rapid, sensitive, specific and cost-effective multiplex assay system will thus prove to be a useful tool to screen a large number of clinical samples. Keeping in view the importance of enteric viruses associated with diseases of public health concern, a multiplex RT-PCR has been developed for detection of nine enteric viruses associated with acute gastroenteritis.

## Methods

### Specimens

Retrospective stool samples (*n*=185) collected earlier from children below 5 years of age hospitalized for acute gastroenteritis in local hospitals of Pune (Maharashtra) India, were selected for testing nine different enteric viruses (group A rotavirus, norovirus GG II, sapovirus, adenovirus, astrovirus, enterovirus, human parechovirus, aichivirus and bocavirus) by newly developed RT-multiplex PCR assay. *The clinical symptoms of the patients included diarrhea with* *three* *episodes within a* *24* *h* *duration with or without fever and vomiting* *.* All these samples were previously analysed for the presence of nine different enteric viruses by monoplex PCR and confirmed positive for respective viruses.

### Ethical clearance

The research work has been carried out after obtaining approval from the Institutional Human Ethical Committees (IHEC) of National Institute of Virology, Pune. Informed consent was obtained from the parents/guardians prior to case registration and specimen collection.

### Nucleic acid extraction

Thirty percent (30% w/v) faecal suspension was prepared using 1× PBS (pH 7.2). RNA extraction was carried out by commercially available 5× Mag Max 96 viral isolation kit (Ambion, USA).

### Primers

Published primers for nine different enteric viruses were used for the amplification of different genomic regions such as the VP6 gene for rotavirus [20], Hexon region for adenovirus [21], ORF-1 for astro [22] and RdRp for sapovirus [23], 5′NCR for enterovirus [24], 3C/3D for aichi virus [25], 5′UTR for human parechovirus [26] , VP1/2 for bocavirus [27] and RdRp for norovirus GG II [28]. Primer sequences and nucleotide position are mentioned in Table 1.

**Table 1.** Specific primers used in multiplex RT-PCR for amplifying the nine enteric virus genomes (AiV, SaV, HPeV, RV, AstV, HBoV, EVs, AdV and NoV viruses) in acute gastroenteritis specimens

**Viruses and primers**	**Target region**	**Sequence 5–** **3**	**Sense**	**Position**	**Amplicon size (bp**)	**Reference**
Aichi virus 6261 6779	3 CD	ACACTCCCACCTCCCGCCAGTA GGAAGAGCTGGGTGTCAAGA	+ −	6261–6282 6779–6760	519 bp	[25]
Sapo Virus SV F22 SV-R2	ORF-1	SMWAWTAGTGTTTGARATG GWGGGRTCAACMCCWGGTGG	+ −	5154–5172 5591–5177	420 bp	[23]
Human Parecho PE-5F PE-5R	5′UTR	CCACGCTYGTGGAYCTTATG GGCCTTACAACTAGTGTTTGC	+ −	292–312 553–533	261 bp	[26]
Boca virus AK-VP-F2 AK-VP-R2	VP1/2	GGCTCCTGCTCTAGGAAATAAAGAG CCTGCTGTTAGGTCGTTGTTGTATGT	+ −	3233–3257 3779–3805	575 bp	[27]
Rotavirus VP6-F VP6-R	VP6	GACGGVGCRACTACATGGT GTCCAATTCATNCCTGGTGG	+ −	7 747–766 1106–1126	379 bp	[20]
Astrovirus Mon340-F Mon348-R	ORF 1a	CGTCATTATTTGTTGTCATACT ACATGTGCTGCTGTTACTATG	+ −	1182–1204 1470–1449	289 bp	[22]
Entero virus 160 F 597 R	5′NCR	CAAGCACTTCTGTTTCCCCGG ATTGTCACCATAAGCAGCCA	+ −	160–180 599–580	440 bp	[24]
Adeno virus Hexon1deg Hexon2deg	Hexon	GCC SCA RTG GKC WTA CAT GCA CAT C CAG CAC SCC ICG RAT GTC AAA	+ −	21–46 322–301	301 bp	[21]
Noro virus SR46-F GR12-R	RdRp	TGGAATTCCATCGCCCACTGG AGTTGTCACGATCTCATCATCACC	+ −	4754–4775 4879–4853	125 bp	[28]

Y means C or T; W means A or T; and R means A or G; S means G or C; M means A or C; N means Any base; V means A,C or G; I means Inosine).

### Multiplex PCR

Detection of three different enteric viruses in a single tube (tube 1: aichivirus, sapovirus and human parechovirus; tube 2: bocavirus, rotavirus and astrovirus; tube 3: enterovirus, adenovirus and norovirus GG II virus) was carried out using One Step RT-PCR kit available commercially (Qiagen, Hilden, Germany).

Briefly, 4 μl of the extracted RNA was used for RT by denaturing at 94 °C for 3 min and then chilled on ice for 2 min. A reaction mix of 21 μl containing 5 μl of 5× buffer, 1 μl of dNTPs (10 mM), Rnase-free water, 1 μl/0.3 μl/0.2 μl each of 20 μM of three pairs of specific primers for tube 1/tube 2/tube 3 and 1 μl of the enzyme mix were added to make up the final volume to 25 μl. The reaction tubes were placed in a thermal cycler for 30 min at 45 and 95 °C for 15 min followed by 40 cycles of 94 °C for 1 min, 45 °C/50 °C for 1 min, 70 °C for 2.5 min with a final extension carried at 70 °C for 7 min. Annealing temperature was set at 45 and 50°C and was used for tube 1, tube 2 and for tube 3 viruses, respectively.

### Monoplex PCR

Detection of single enteric virus was carried out by monoplex PCR using the same protocol as described above for the multiplex PCR. Except 1 μl each of specific primers (20 μm) was used in the reaction mixture [20–28].

### Positive virus controls

The faecal specimens tested positive for a single virus (rota/noro GG II /enteric adeno/astro/aichi/sapo/boca/entero virus) using One-Step RT-PCR kit were selected for further standardization of multiplex PCR.

### Negative virus controls

Distilled water and stool samples tested negative for all nine enteric viruses served as negative controls.

### Specificity testing of the three primer pairs

Specificity of the test mixture of three primer pairs was determined using appropriate positive controls. PCR mixture containing three primer pairs and each single target template (RNA), and three primer pairs and three target templates was conducted in two different combinations. Specificity of the test was also carried out using faecal samples negative for the target virus but positive for other viruses.

### Electrophoresis

All the PCR products were analysed by horizontal gel electrophoresis using 1× Tris acetate EDTA (TAE) buffer pH 8.3 on 2% agarose gels, containing ethidium bromide (0.5 mg ml^−1^) and visualized under UV transilluminator (AlfaImager HP Imaging System, CA, USA).

### Sequence analysis

The PCR products generated by multiplex RT-PCR were purified and subjected to cycle sequencing to confirm the amplicons of target viral sequences.

### Sensitivity and specificity testing of the assay

For this purpose a total of 230 faecal samples were selected, which included 45 samples collected from non-diarrheal cases and tested negative for all nine enteric viruses as negative controls. All 185 clinical samples collected from diarrheal cases tested positive earlier by monoplex PCR for one of the nine enteric viruses served as the positive controls.

## Results

### Detection of target viruses in faecal specimens by multiplex RT-PCR

Retrospective faecal specimens (*n*=185) collected from children hospitalized for acute gastroenteritis were tested for all nine enteric viruses by monoplex PCR. Of these positivity for a single virus was confirmed in 101 (54.6%) of the samples [rotavirus (*n*=84), astrovirus (*n*=4), enterovirus (*n*=10), adenovirus (*n*=1), norovirus GG II (*n*=2)]. Mixed infections with AiV/SV/HPeV/BoV/RV were detected in 84 (45.4%) of the samples. All these 185 clinical samples tested by monoplex PCR were subjected to currently developed RT-multiplex PCR assay in order to establish the concordance of these two assay systems.

Based on this approach, results showed 98.9% (183/185) concordance between monoplex PCR and multiplex assay. Of the 183 positive samples 54.6% (*n*=101) of them showed single infections with rotavirus (*n*=84), astro (*n*=4), entero (*n*=10) and noro (*n*=2) viruses. The remaining 82 (44.8%) samples showed mixed infections with two or three viruses. These included mixed infections with rotavirus (*n*=61) followed by enterovirus (*n*=15), astrovirus (*n*=13), adenovirus (*n*=20), human parechovirus (*n*=10) and two each in sapovirus and norovirus. Overall, predominance of group A rotavirus (139/183, 75.9%) followed by enterovirus (11.47%, 21), astrovirus (4.37%, 8), adenovirus (2.7%, 5), norovirus GII (1.64%, 3) were observed and two of the samples tested negative in the assay. *Although, the* *n* *oroviruses detected in the study belonged to* *g* *enogroup II, no genogroup I strains were detected.*

Of the 124 mixed infections, predominance of RV mixed infections was observed in 61 (49.2%) followed by HPeV in 33 (26.6%), AiV in 31 (25%), BoV in 28 (22.6%), EV in 16 (13%), AdV in 20 (16.1%), AstV in 13 (10.5%) and 2 (1.6%) in NoV and SaVs (Table 2). Combinations between group A rotavirus and human bocavirus in 19 (31.1%), parechovirus in 16 (26.2%), aichivirus in 15 (24.6%), enterovirus in 10 (16.4%) and Sapovirus in 1 (1.6%) were detected (Table 2). Mixed infections of different non-rota enteric viruses were detected as mentioned; EV and AiV or BoVs or HPeV were found in three, five and seven of the clinical samples each. AdV was co-detected with SaV (*n*=1), HPeV (*n*=7), AiV (*n*=2) and RV and EV (*n*=5) each AstV was co-detected with AdV (*n*=6), AiV (*n*=2), with SaV, HPeV, EV, BoV and RV in one sample each (Table 2).

**Table 2.** Combination of different enteric viruses in mixed infections detected by multiplex PCR in diarrheal cases

Enteric viruses	Enteric viruses detected by multiplex PCR								
	AiV (*n*)	NoV (*n*)	EV (*n*)	BoV (*n*)	RV (*n*)	SaV (*n*)	HPeV (*n*)	AstV (*n*)	AdV (*n*)
**AiV (31**)	–	0	3	1	15	2	6	2	2
**SaV (4**)	–	0	0	0	1	–	1	1	1
**BoV(28**)	–	0	5	–	19	–	3	1	0
**HPeV (33**)	–	2	7	–	16	–	–	1	7
**EV (16**)	–	0	–	–	10	–	–	1	5
**NoV (0**)	–	–	–	–	–	–	–	–	–
**AdV(1**)	–	0	–	–	0	–	–	1	–
**RV (11**)	–	–	–	–	–	–	–	6	5
**AstV(0**)	–	–	–	–	–	–	–	–	–
**Total (124**)	**0**	**2**	**15**	**1**	**61**	**2**	**10**	**13**	**20**

Twenty-three clinical samples (23/82; 28%) showed mixed infections with more than two enteric viruses. Of these, 17 specimens (17/23, 73.9%) showed group A RV infections in combination with other enteric viruses.

### Specificity testing of the three primer pairs

**Fig. 1.**
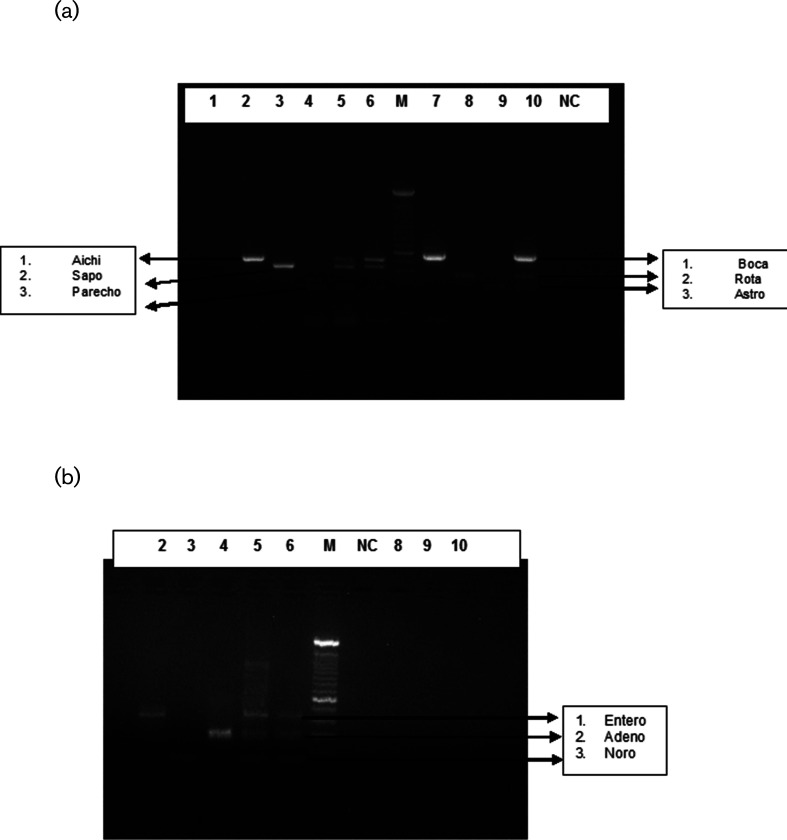
(a) Specificity testing of (tubes 1 and 2) the multiplex RT-PCR assay with a mixture of three primer pairs for three positive controls. Lanes 2–4, aichi, sapo and HPeV, respectively. Lanes 5 and 6 the mixture of aichi virus, sapo and HPeV positive controls, M, marker. Lanes 7–9, boca, rota and astro, respectively. Lane 10 the mixture of bocavirus, rota and astro positive controls. (b) Specificity testing of the (tube 3) multiplex PCR assay with a mixture of three primer pairs for three positive controls. Lanes 2–4, entero, noro and adeno, respectively. Lanes 5 and 6, the mixture of entero, noro and adeno positive controls; M, marker.

The specificity of the test was conducted using positive controls in combination with the three primer pairs used in a single tube (Fig. 1). Combination of primers used in the study had amplified the viral genomes of the positive controls and generated three different expected size amplicons, i.e. 519, 420 and 261 bp for tube 1 viruses (aichivirus, sapovirus and human parechovirus), 575, 379 and 289 bp for tube 2 viruses (bocavirus, rotavirus and astrovirus) and 437, 301 and 125 bp for tube 3 viruses (enterovirus, adenovirus and norovirus GGII), respectively, in 2% agarose gel electrophoresis. Cycle sequencing of the individual virus amplicons was carried out in order to show its authenticity (data shown in Supplement 1, available in the online version of this article). No cross-reaction with non-targets was noticed. Negative controls did not show any amplification.

### Sensitivity and specificity

Control samples (*n*=230), which included negative controls (*n*=45) and positive controls (*n*=185) when tested by currently developed multiplex RT-PCR assay, showed 98.9% sensitivity and 97.7% specificity and the results have been appended in Table 3.

**Table 3.** Sensitivity and specificity of the multiplex RT-PCR assay using control samples

Samples	Positive	Negative	Total
Positive (*n*=185)	183	2	185
Negative (*n*=45)	1	44	45

Sensitivity: 98.9%

Specificity: 97.7%

## Discussion

Globally diarrheal disease is considered as a major cause of mortality among children, and enteric viruses play a major etiologic role in causing gastroenteritis with incidence ranging from 45–86% [29–32]. It is mandatory to understand the possible viral etiological agents associated with diarrhea for implementation of interventions. The availability of affordable viral diagnosis will further help to improve patient care by reducing the unnecessary use of antibiotics [33]. Currently, molecular diagnosis by RT-PCR-based tool is considered to be a convenient, useful and powerful approach for detecting enteric viral pathogens from clinical specimens. It is also widely used as this assay provides simple, rapid and cost-effective laboratory diagnosis [34].

A sensitive multiplex PCR assay was developed to detect nine different enteric viruses simultaneously in faecal specimens collected from diarrheal patients. Although the assay included both DNA/RNA viruses, the same protocol standard of RT-PCR was used for detection of these viruses. This was done to minimize the handling and contamination problems. Also, the results could be co-related by using the same RNA and/or DNA as a template. The results demonstrated that the newly developed multiplex RT-PCR was specific in detecting nine enteric viruses and no cross-reaction with non-targets was observed (Fig. 1). The overall results showed good concordance (98.9%) in viral detection rates between monoplex RT-PCR and multiplex RT-PCR carried out on clinical specimens from diarrheal cases.

Studies carried out earlier, showed the presence of eight viral agents in faecal specimens as determined by two sets of primers used in multiplex RT-PCR [14, 15]. Subsequently, a multiplex PCR method using multiple primer pairs was used to detect four and ten viruses simultaneously [7, 17]. In India, although there have been few reports available on monoplex PCR for detection of enteric viruses in acute gastroenteritis cases, so far no such attempts have been made to detect a wide range of enteric viruses using One-Step RT-PCR kit. Also in contrast to the other studies reported, the combinations and panel of viruses selected in the currently developed multiplex PCR and the methodology optimized was completely different [7, 17, 35]. The sensitivity was also increased by using the MagMax RNA extraction method followed by multiplex PCR using the Qiagen One-Step RT-PCR kit as described earlier [36]. The sensitivity and specificity of the newly developed RT-multiplex PCR demonstrated a good concordance (98.9%) with the monoplex PCR. The primer set used in this study was appropriate for screening nine different enteric viruses in clinical specimens from infants and children with acute gastroenteritis. The same primers had been used earlier in conventional PCRs for the detection of single infections [20–28].

When all the faecal specimens (*n*=185) tested positive for different enteric viruses by monoplex RT-PCR were selected for multiplex PCR assay, high percentage of mixed infections (84/185, 46%) were detected. These results were in good agreement to the reports available recently from Burkina Faso, 35.7% [30] and China, 23% [37]. Human parechovirus and bocavirus have been known to cause sepsis-like illness with meningitis and respiratory tract infections [12, 38] in young infants and children. Frequent detection and the high prevalence of these viruses in faecal specimens of children with acute gastroenteritis (AGE) worldwide have raised the possibility of their association with viral gastroenteritis [5, 39, 40].

Co-infections of rotavirus and bocavirus or parechovirus were noted at 26.2–31.1% level by this method. These findings differed from those reported from the USA, where 3.95–5.26% prevalence in AGE cases was reported by using the monoplex PCR for these viruses [41]. Earlier reports have suggested that mixed infections may be responsible for more-severe diarrhea [42, 43]. The possible explanation for this could be that RVA infections cause enterocyte destruction from the top of intestinal villus, which increases the risk of opportunistic pathogen infection [41].

Since it is known that diarrhea is often caused due to multiple enteric pathogens [44, 45], it is mandatory to screen diarrheal samples for the most prevalent pathogens using a rapid, sensitive, cost effective assay such as a multiplex PCR. Such a type of assay system will be especially useful while handling a large number of clinical samples during diarrheal epidemics and for conducting outbreak investigations of gastroenteritis where results need to be communicated to clinicians in a short span of time. However, laboratory investigations conducted using molecular techniques needs to be compared with clinical symptoms/history of the patients to find out the actual causative agent [46, 47] especially when mixed infections are detected. In this study, detection of other enteric viruses such as bocavirus and parechoviruses associated with diarrheal diseases has also been included as recent studies have shown the prevalence and importance of these viruses [48, 49]. Also, the possibilities of asymptomatic colonization cannot be ruled out.

In summary, the currently developed multiplex RT-PCR assay would be useful in screening nine different enteric viruses in the clinical laboratory. Outcome of the approach would be helpful in patient care, clinical management and for development of intervention strategies as diarrheal disease is a major global public health problem.

## Supplementary Data

Supplementary material 1
