# Supplementary material for: Detection of different enteric viruses in children with diarrheal disease: evidence of the high frequency of mixed infections
Source: Access Microbiol. 2019 Mar 29;1(2):e000010. doi: 10.1099/acmi.0.000010 (PMC7470349; doi:10.1099/acmi.0.000010)
Supplement: Supplementary material 1 [file acmi-1-010-s001.pdf]

Norovirus Standard

Nucleotide Sequence (180 letters)

RID H7BJX7F201R (Expires on 03-27 14:59 pm)

Query ID lcl|Query_231431
Description None
Molecule type nucleic acid
Query Length 180

Database Name nr
Description Nucleotide collection (nt)
Program BLASTN 2.2.31+ Citation

Other reports: Search Summary [Taxonomy reports] [Distance tree of results]

Graphic Summary

Distribution of 100 Blast Hits on the Query Sequence

Mouse over to see the defline, click to show alignments

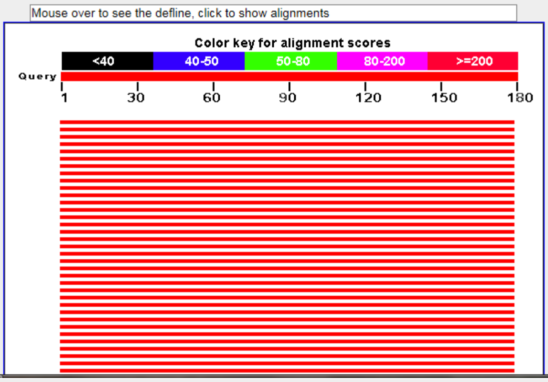

Sequences producing significant alignments:

Select: All None Selected:0

Alignments Download GenBank Graphics Distance tree of results

	Description	Max score	Total score	Query cover	E value	Ident	Accession
☐	Norovirus Hu/GII.4/Riviera1590/2008/US polymerase gene, partial cds; and VP1 and VP2 genes, complete cds	331	331	99%	2e-87	100%	GQ850882.1
☐	Norovirus GII isolate GII/Hu/ESP/2009/GII.Pe_GII.4/SanSebastian255099 RNA dependent RNA polymerase and major capsid protein genes, partial cds	326	326	99%	9e-86	99%	KJ504413.1
☐	Norovirus Hu/GII/BCHN34/2008/IND genes for RNA-dependent RNA polymerase, capsid protein, partial cds	326	326	99%	9e-86	99%	AB592967.1
☐	Norovirus Hu/GII/IDH1478/2009/IND genes for RNA-dependent RNA polymerase, capsid protein, partial cds	326	326	99%	9e-86	99%	AB592959.1
☐	Norovirus Hu/GII/US/230107-2/2007/SGP capsid gene, partial cds	326	326	99%	9e-86	99%	GQ925167.1
☐	Norovirus Hu/V1783/07/IND RdRp, VP1 genes for RNA dependent RNA polymerase, capsid protein, partial cds	326	326	99%	9e-86	99%	AB447426.1
☐	Norovirus Hu/V1776/07/IND RdRp, VP1 genes for RNA dependent RNA polymerase, capsid protein, partial cds	326	326	99%	9e-86	99%	AB447425.1
☐	Norovirus Hu/V1774/07/IND RdRp, VP1 genes for RNA dependent RNA polymerase, capsid protein, partial cds	326	326	99%	9e-86	99%	AB447424.1
☐	Norovirus Hu/V1766/07/IND RdRp, VP1 genes for RNA dependent RNA polymerase, capsid protein, partial cds	326	326	99%	9e-86	99%	AB447422.1
☐	Norovirus Hu/V1760/07/IND RdRp, VP1 genes for RNA dependent RNA polymerase, capsid protein, partial cds	326	326	99%	9e-86	99%	AB447421.1
☐	Norovirus Hu/V1750/07/IND RdRp, VP1 genes for RNA dependent RNA polymerase, capsid protein, partial cds	326	326	99%	9e-86	99%	AB447420.1
☐	Norovirus Hu/V1749/07/IND RdRp, VP1 genes for RNA dependent RNA polymerase, capsid protein, partial cds	326	326	99%	9e-86	99%	AB447419.1
☐	Norovirus Hu/OC07138/07/JP genes for RNA-dependent RNA polymerase, capsid protein, partial and complete cds	326	326	99%	9e-86	99%	AB434770.1
☐	Norovirus GII isolate Hu/ZA/2011/GII.4/Osaka 2007/Cape Town 8179 RNA-dependent RNA polymerase gene, partial cds; and capsid protein gene, complete cds	320	320	99%	4e-84	99%	KM025143.1
☐	Norovirus Hu/GII/10148/2009/VNM nonstructural polyprotein and capsid protein VP1 genes, complete cds; and capsid protein VP2 gene, partial cds	320	320	99%	4e-84	99%	KM198485.1
☐	Norovirus Hu/GII.4/A/GBR/2010 clone PxA120710, partial genome	320	320	99%	4e-84	99%	KC810028.1
☐	Norovirus Hu/GII.4/Armidale/NSW390I/2008/AU, complete genome	320	320	99%	4e-84	99%	GQ845369.3
☐	Norovirus Hu/GII/IDH1064/2008/IND genes for RNA-dependent RNA polymerase, capsid protein, partial cds	320	320	99%	4e-84	99%	AB592968.1
☐	[illegible]	[illegible]	[illegible]	[illegible]	[illegible]	[illegible]	[illegible]

Astrovirus Std

Nucleotide Sequence (245 letters)

RID H7BPGWKH01R (Expires on 03-27 15:01 pm)
Query ID lcl|Query_261083
Description None
Molecule type nucleic acid
Query Length 245

Database Name nr
Description Nucleotide collection (nt)
Program BLASTN 2.2.31+ Citation

Other reports: Search Summary [Taxonomy reports] [Distance tree of results]

Graphic Summary

Distribution of 100 Blast Hits on the Query Sequence

Mouse over to see the define, click to show alignments

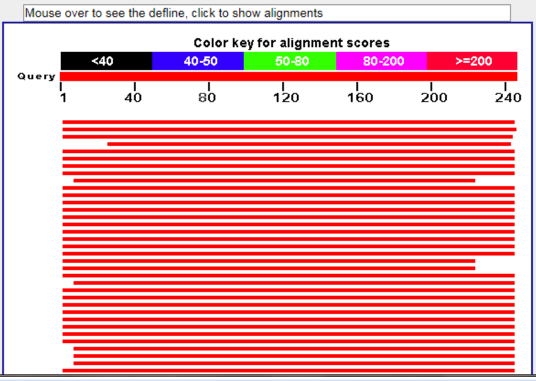

Sequences producing significant alignments:

Select: All None Selected:0

Alignments Download GenBank Graphics Distance tree of results

	Description	Max score	Total score	Query cover	E value	Ident	Accession
☐	Human astrovirus 5 isolate DL030, complete genome	420	420	98%	6e-114	98%	JQ403108.1
☐	Mamastrovirus 1 strain PAK_NIH_VS_908 capsid protein gene, partial cds	399	399	99%	7e-108	96%	KF453630.1
☐	Human astrovirus type 4 strain Dresden, complete genome	396	396	98%	9e-107	96%	AY720891.1
☐	Human astrovirus 3a strain MAstV/Hu/BG/2009/BG656 nonstructural protein (ORF1a) gene, partial cds	388	388	88%	2e-104	99%	KP162248.1
☐	Mamastrovirus 1 gene for serine protease, partial cds, isolate: IDH2211, primer pair MON340 and P6	387	387	98%	6e-104	95%	AB639820.1
☐	Human astrovirus serotype 2, complete sequence of genomic RNA	387	387	98%	6e-104	95%	L13745.1
☐	Human astrovirus clone KS106207 non-structural protein mRNA, partial cds	364	364	98%	3e-97	94%	AF361032.1
☐	Human astrovirus clone KS106205 non-structural protein mRNA, partial cds	359	359	98%	1e-95	93%	AF361030.1
☐	Human astrovirus 5 isolate IRAN/AHVAZ5/2010 non-structural protein gene, partial cds	353	353	87%	6e-94	96%	JX171285.1
☐	Mamastrovirus 1 ORF1 gene for nonstructural protein, complete cds, isolate: V1182	348	348	98%	3e-92	93%	AB325804.1
☐	Mamastrovirus 1 Orf1a gene for nonstructural protein, partial cds, isolate: V1182, PCR_primers: fwd_name: MON340, rev_name: P6	348	348	98%	3e-92	93%	AB303397.1
☐	Human astrovirus 1 Beijing/347/2007/CHN nonstructural protein gene, partial cds	342	342	98%	1e-90	92%	FJ755363.1
☐	Human astrovirus 1 Beijing/234/2006/CHN nonstructural protein gene, partial cds	342	342	98%	1e-90	92%	FJ755355.1
☐	Human astrovirus 1 Beijing/210/2006/CHN nonstructural protein gene, partial cds	342	342	98%	1e-90	92%	FJ755354.1
☐	Human astrovirus 1 Beijing/189/2005/CHN nonstructural protein gene, partial cds	342	342	98%	1e-90	92%	FJ755344.1
☐	Human astrovirus 1 Beijing/46/2005/CHN nonstructural protein gene, partial cds	342	342	98%	1e-90	92%	FJ755339.1
☐	Human astrovirus 1 Beijing/293/2007/CHN, complete genome	342	342	98%	1e-90	92%	FJ755405.1
☐	Human astrovirus clone KS106208 non-structural protein mRNA, partial cds	342	342	98%	1e-90	92%	AF361033.1
☐	[illegible]	[illegible]	[illegible]	[illegible]	[illegible]	[illegible]	[illegible]

Adenovirus Std

Nucleotide Sequence (246 letters)

RID H7C9XE1W01R (Expires on 03-27 15:11 pm)

Query ID lcl|Query_46941
Description None
Molecule type nucleic acid
Query Length 246

Database Name nr
Description Nucleotide collection (nt)
Program BLASTN 2.2.31+ Citation

Other reports: Search Summary [Taxonomy reports] [Distance tree of results]

Graphic Summary

Distribution of 100 Blast Hits on the Query Sequence

Mouse over to see the define, click to show alignments

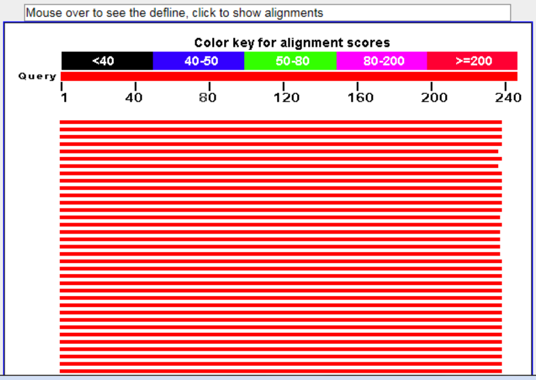

Sequences producing significant alignments:

Select: All None Selected:0

Alignments Download GenBank Graphics Distance tree of results

Description	Max score	Total score	Query cover	E value	Ident	Accession
Human adenovirus 41 isolate CR8359 hexon gene, partial cds	429	429	96%	9e-117	99%	KC953658.1
Human adenovirus 41 isolate Q837 hexon gene, partial cds	429	429	96%	9e-117	99%	KF669135.1
Human adenovirus 41 isolate Q513 hexon gene, partial cds	429	429	96%	9e-117	99%	KF669124.1
Human adenovirus 41 isolate CR7535 hexon gene, partial cds	429	429	96%	9e-117	99%	JX412907.1
Human adenovirus 41 isolate CR7538 hexon gene, partial cds	425	425	95%	1e-115	99%	JX412909.1
Human adenovirus 41 isolate CR7513 hexon gene, partial cds	425	425	96%	1e-115	99%	JX412904.1
Human adenovirus 41 isolate CR6780 hexon gene, partial cds	425	425	95%	1e-115	99%	JX412889.1
Human adenovirus 41 isolate CR6711 hexon gene, partial cds	425	425	96%	1e-115	99%	JX412878.1
Human adenovirus 41 isolate 10-4851, complete genome	424	424	96%	4e-115	99%	KF303071.1
Human adenovirus 41 isolate NY/2010/4845, complete genome	424	424	96%	4e-115	99%	KF303070.1
Human adenovirus 41 isolate NY/2010/4849, complete genome	424	424	96%	4e-115	99%	KF303069.1
Human adenovirus 41 isolate CR8233 hexon gene, partial cds	424	424	96%	4e-115	99%	KC953649.1
Human adenovirus 41 DNA, complete genome, strain: SaP3-3F	424	424	96%	4e-115	99%	AB728839.1
Human adenovirus 41 isolate Q1046 hexon gene, partial cds	424	424	96%	4e-115	99%	KF669141.1
Human adenovirus 41 isolate Q632 hexon gene, partial cds	424	424	96%	4e-115	99%	KF669130.1
Human adenovirus 41 isolate Q579 hexon gene, partial cds	424	424	96%	4e-115	99%	KF669127.1
Human adenovirus 41 isolate Q575 hexon gene, partial cds	424	424	96%	4e-115	99%	KF669125.1
Human adenovirus 41 isolate Q510 hexon gene, partial cds	424	424	96%	4e-115	99%	KF669123.1
[illegible]	[illegible]	[illegible]	[illegible]	[illegible]	[illegible]	[illegible]

Group A Rotavirus Std

Nucleotide Sequence (326 letters)

RID H7BA3CNB01R (Expires on 03-27 14:54 pm)

Query ID lcl|Query_149741
Description None
Molecule type nucleic acid
Query Length 326

Database Name nr
Description Nucleotide collection (nt)
Program BLASTN 2.2.31+ Citation

Other reports: Search Summary [Taxonomy reports] [Distance tree of results]

Graphic Summary

Distribution of 100 Blast Hits on the Query Sequence

Mouse over to see the defline, click to show alignments

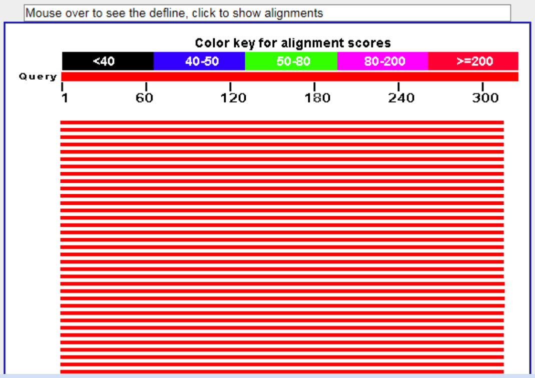

p://blast.ncbi.nlm.nih.gov/Blast.cgi#631919719

Sequences producing significant alignments:

Select: All None Selected:0

Alignments Download GenBank Graphics Distance tree of results

Description	Max score	Total score	Query cover	E value	Ident	Accession
Rotavirus A strain RVA/Human-wt/AUS/CK20055/2010/G2P[4] segment 6 inner capsid protein VP6 (VP6) gene, complete cds	562	562	96%	1e-156	99%	KC443204.1
Rotavirus A strain RVA/Human-wt/AUS/CK20048/2011/G2P[4] segment 6 inner capsid protein VP6 (VP6) gene, complete cds	562	562	96%	1e-156	99%	KC443459.1
Rotavirus A strain RVA/Human-wt/SEN/MRC-DPRU1915/2008/G2P[4] segment 6 inner capsid protein VP6 (VP6) gene, complete cds	556	556	96%	6e-155	98%	KJ753527.1
Human rotavirus A strain RVA/Human-wt/HUN/ERN5523/2012/G3P4 VP6 gene, partial cds	556	556	96%	6e-155	98%	KJ919794.1
Rotavirus A isolate RVA/Human/JPN/S13-30/2013/G3P[4] segment 6 VP6 gene, complete cds	556	556	96%	6e-155	98%	KJ639016.1
Human rotavirus A isolate Hu/RUS/O1299/2012/G2P[6] inner capsid protein VP6 (VP6) gene, complete cds	556	556	96%	6e-155	98%	KC713896.1
Rotavirus A strain RVA/Human-wt/ETH/MRC-DPRU2241/2009/G3P[6] segment 6 inner capsid protein VP6 (VP6) gene, complete cds	556	556	96%	6e-155	98%	KJ751893.1
Human rotavirus A isolate Hu/RUS/O1033/2010/G2P[4] inner capsid protein VP6 (VP6) gene, complete cds	556	556	96%	6e-155	98%	KF036162.1
Human rotavirus A isolate Hu/RUS/O1117/2011/G2P[4] inner capsid protein VP6 (VP6) gene, complete cds	556	556	96%	6e-155	98%	KF036165.1
Human rotavirus A isolate Hu/RUS/O805/2011/G2P[4] inner capsid protein VP6 (VP6) gene, complete cds	556	556	96%	6e-155	98%	KF036163.1
Human rotavirus A isolate Hu/RUS/Nov12-N5000/2012/G2P[4] inner capsid protein VP6 (VP6) gene, complete cds	556	556	96%	6e-155	98%	KF036153.1
Human rotavirus A isolate Hu/RUS/Nov12-N5188/2012 inner capsid protein VP6 (VP6) gene, complete cds	556	556	96%	6e-155	98%	KF036155.1
Rotavirus A isolate RVA/Human-wt/USA/VU10-11-24/2011/G2P[4] VP6 gene, complete cds	556	556	96%	6e-155	98%	KF716345.1
Rotavirus A isolate RVA/Human-wt/USA/VU10-11-6/2011/G2P[4] VP6 gene, complete cds	556	556	96%	6e-155	98%	KF716343.1
Rotavirus A isolate RVA/Human-wt/USA/VU10-11-11/2011/G2P[4] VP6 gene, complete cds	556	556	96%	6e-155	98%	KF716340.1
Rotavirus A strain PA108/2007 VP6 mRNA, complete cds	556	556	96%	6e-155	98%	KC178803.1
Rotavirus A strain RVA/Human-wt/AUS/CK20028/2006/G2P[4] segment 6 inner capsid protein VP6 (VP6) gene, complete cds	556	556	96%	6e-155	98%	KC443635.1
Rotavirus A strain RVA/Human-wt/AUS/CK20027/2006/G2P[4] segment 6 inner capsid protein VP6 (VP6) gene, complete cds	556	556	96%	6e-155	98%	KC443767.1
[illegible]	[illegible]	[illegible]	[illegible]	[illegible]	[illegible]	[illegible]

Enterovirus Std

Nucleotide Sequence (329 letters)

RID H7BS7EKS01R (Expires on 03-27 15:02 pm)

Query ID lcl|Query_17809
Description None
Molecule type nucleic acid
Query Length 329

Database Name nr
Description Nucleotide collection (nt)
Program BLASTN 2.2.31+ Citation

Other reports: Search Summary [Taxonomy reports] [Distance tree of results]

Graphic Summary

Distribution of 100 Blast Hits on the Query Sequence

Mouse over to see the defline, click to show alignments

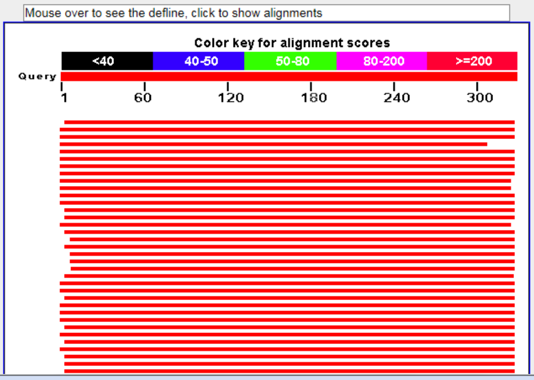

Descriptions

Sequences producing significant alignments:

Select: All None Selected:0

Alignments Download GenBank Graphics Distance tree of results

	Description	Max score	Total score	Query cover	E value	Ident	Accession
☐	Enterovirus B87 isolate LY02/SD/CHN/2000, complete genome	486	486	98%	8e-134	94%	KC292019.1
☐	Human coxsackievirus B1 strain MSH/KM9/2009, complete genome	486	486	99%	8e-134	93%	JN596588.1
☐	Echovirus E30 isolate ECV30/GX10/05, complete genome	481	481	99%	4e-132	93%	JX854435.1
☐	Human echovirus 1 isolate 06.329.0986 5' UTR	481	481	93%	4e-132	95%	GU236152.1
☐	Echovirus E13 gene for polyprotein, complete cds, strain: S3(1)-1	481	481	99%	4e-132	93%	AB501332.1
☐	Echovirus E13 gene for polyprotein, complete cds, strain: 2002-240-SF	481	481	99%	4e-132	93%	AB501329.1
☐	Human coxsackievirus B1 isolate CVB1SD2011CHN, complete genome	475	475	99%	2e-130	93%	JX976769.1
☐	Human echovirus 30 strain Kor08-ECV30, complete genome	475	475	99%	2e-130	93%	JN704615.1
☐	Echovirus E13 gene for polyprotein, complete cds, strain: I5(1)-1	475	475	98%	2e-130	93%	AB501331.1
☐	Echovirus E13 gene for polyprotein, complete cds, strain: 2002-245-NP	475	475	98%	2e-130	93%	AB501330.1
☐	Human echovirus 19 strain Djum/91 5' non-translated region, partial sequence	473	473	99%	6e-130	93%	AF447480.1
☐	Human coxsackievirus B3 strain Fuyang19, complete genome	470	470	99%	8e-129	92%	FJ000001.1
☐	Human enterovirus 84 isolate CIV2003-10603, complete genome	470	470	98%	8e-129	93%	DQ902712.1
☐	Human echovirus 11 strain Kar/87 polyprotein gene, complete cds	470	470	98%	8e-129	93%	AY167104.1
☐	Human coxsackievirus B5 isolate 2000/CSF/KOR, complete genome	470	470	98%	8e-129	93%	AY875692.1
☐	Human coxsackievirus B3 strain 2679, complete genome	468	468	98%	3e-128	93%	KJ489414.1
☐	Human echovirus 6 strain FJLY2010327, complete genome	468	468	97%	3e-128	93%	JQ729993.1
☐	Human enterovirus B isolate 2006-Kwater-EV-10 5' UTR	468	468	98%	3e-128	93%	EU252012.1
☐	[illegible]	[illegible]	[illegible]	[illegible]	[illegible]	[illegible]	[illegible]

HumanParechovirus Std

Job title: Nucleotide Sequence (203 letters)

RID 0WEGZ3BM014 (Expires on 12-11 17:04 pm)

Query ID lcl|Query_235393
Description None
Molecule type nucleic acid
Query Length 203

Database Name nr
Description Nucleotide collection (nt)
Program BLASTN 2.8.1+ Citation

Other reports: Search Summary [Taxonomy reports] [Distance tree of results] [MSA viewer]

Graphic Summary

Distribution of the top 100 Blast Hits on 100 subject sequences

Mouse over to see the title, click to show alignments

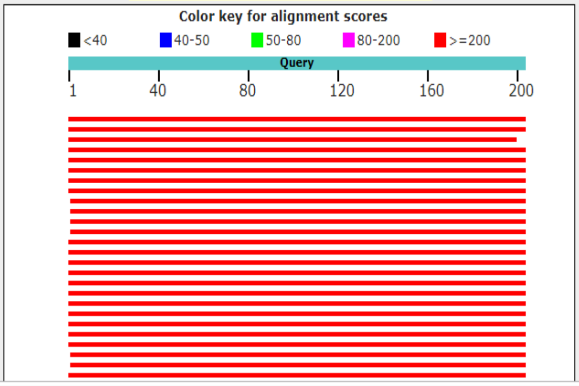

Questions/comments

Alignments Download GenBank Graphics Distance tree of results

	Description	Max score	Total score	Query cover	E value	Ident	Accession
	Human parechovirus 1 strain 1917/N.Nov/RU/2008 5' UTR	343	343	100%	3e-90	98%	JQ437880.1
	Human parechovirus 1 strain 14990/N.Nov/RU/2007 5' UTR	338	338	100%	4e-89	97%	JQ437877.1
	Human parechovirus 1 strain 1994/N.Nov/RU/2008 5' UTR	335	335	98%	5e-88	98%	JQ437882.1
	Human parechovirus strain USA/TN/2015-OB2038, complete genome	334	334	100%	2e-87	97%	KY271948.1
	Human parechovirus 1 isolate CAU10-NN, complete genome	334	334	100%	2e-87	97%	JX575746.1
	Human parechovirus 1 strain 14191/N.Nov/RU/2006 5' UTR	334	334	100%	2e-87	97%	JQ437875.1
	Human parechovirus 1, complete genome	329	329	100%	2e-86	96%	FM178558.1
	Human parechovirus 1 isolate BNI-788St, complete genome	329	329	100%	2e-86	96%	EF051629.2
	Human parechovirus 4 clone V2A polyprotein gene, partial cds	327	327	99%	7e-86	96%	MG571807.1
	Human parechovirus 4 isolate FI121301 polyprotein gene, complete cds	327	327	99%	7e-86	96%	KY404171.1
	Human parechovirus 4 isolate FI121290 polyprotein gene, complete cds	327	327	99%	7e-86	96%	KY404170.1
	Human parechovirus 4 isolate K251176-02, complete genome	327	327	99%	7e-86	96%	DQ315670.1
	Human parechovirus 1 isolate ETH_P28_2016 polyprotein gene, complete cds	324	324	100%	8e-85	96%	MG026490.1
	Human parechovirus 1 isolate ETH_P5_2016 polyprotein gene, complete cds	324	324	100%	8e-85	96%	MG026486.1
	Human parechovirus 1 strain 16-G4, complete genome	324	324	100%	8e-85	96%	KY645965.1
	Human parechovirus 1 strain 16-G10, complete genome	324	324	100%	8e[illegible]	[illegible]	[illegible]
	Human parechovirus 1 isolate 146Chzi02, complete genome	324	324	100%	8e[illegible]	[illegible]	[illegible]

Questions/comments

Bocavirus Std

Job title: Nucleotide Sequence (475 letters)

RID 0WHT03Y6015 (Expires on 12-11 17:59 pm)

Query ID lcl|Query_243923
Description None
Molecule type nucleic acid
Query Length 475

Database Name nr
Description Nucleotide collection (nt)
Program BLASTN 2.8.1+ Citation

Other reports: Search Summary [Taxonomy reports] [Distance tree of results] [MSA viewer]

Graphic Summary

Distribution of the top 100 Blast Hits on 100 subject sequences

Mouse over to see the title, click to show alignments

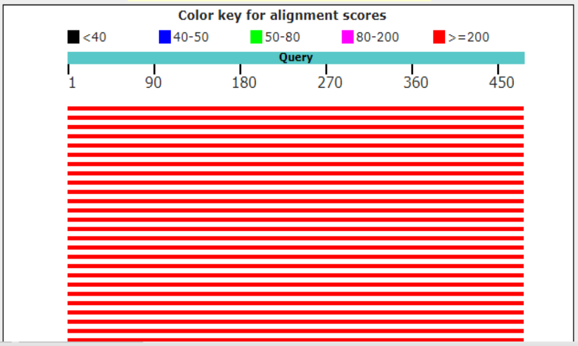

Questions/comments

	Description	Max score	Total score	Query cover	E value	Ident	Accession
☐	Human bocavirus isolate HBoV/ACRI_0111/USA/2016, partial genome	790	790	99%	0.0	97%	MF314144.1
☐	Human bocavirus strain Hu/BRA/2005/HBoV-1/RJ_LVCA11670 VP1 gene, partial cds	790	790	99%	0.0	97%	MF034125.1
☐	Human bocavirus strain Hu/BRA/2005/HBoV-1/RJ_LVCA10968 VP1 gene, partial cds	790	790	99%	0.0	97%	MF034123.1
☐	Human bocavirus 1 strain Hu/BRA/2009/HBoV-1/RJ_LVCA16675 VP1 gene, partial cds	790	790	99%	0.0	97%	KY882312.1
☐	Human bocavirus 1 strain Hu/BRA/2001/HBoV-1/RJ_LVCA4544 VP1 gene, partial cds	790	790	99%	0.0	97%	KY882304.1
☐	Human bocavirus 1 strain Hu/BRA/1999/HBoV-1/RJ_LVCA2502 VP1 gene, partial cds	790	790	99%	0.0	97%	KY882299.1
☐	Human bocavirus 1 strain Hu/BRA/1997/HBoV-1/RJ_LVCA961 VP1 gene, partial cds	790	790	99%	0.0	97%	KY882298.1
☐	Primate bocaparvovirus 1 isolate HBoV1s6 nonstructural protein (NP1) gene, complete cds; and capsid protein (VP1/VP2) gene, partial cds	790	790	99%	0.0	97%	KY629423.1
☐	Primate bocaparvovirus 1 isolate HBoV1m6, complete genome	790	790	99%	0.0	97%	KY629421.1
☐	Human bocavirus isolate Eg/BSU-3, complete genome	790	790	99%	0.0	97%	KU557406.1
☐	Human bocavirus isolate Eg/BSU-2, complete genome	790	790	99%	0.0	97%	KU557405.1
☐	Human bocavirus 1 isolate Pune/NIV1018816/2010/India capsid protein VP1 (VP1) gene, partial cds	790	790	99%	0.0	97%	KU667142.1
☐	Human bocavirus 1 isolate Pune/NIV0912860/2009/India capsid protein VP1 (VP1) gene, partial cds	790	790	99%	0.0	97%	KU667128.1
☐	Human bocavirus 1 strain Pa65-Apr2014 VP1 (VP1) and VP2 (VP2) genes, complete cds	790	790	99%	0.0	97%	KR014468.1
☐	Human bocavirus 1 strain Pa67-Apr2014 VP1 (VP1) and VP2 (VP2) genes, complete cds	790	790	99%	0.0	97%	KR014467.1
☐	Human bocavirus 1 strain 286-Jan2012 VP1 (VP1) and VP2 (VP2) genes, complete cds	790	790	99%	0.0	97%	KR014461.1
☐	Human bocavirus 1 strain 276-Jan2012 VP1 (VP1) and VP2 (VP2) genes, complete cds	790	790	99%	0.0	97%	KR014460.1
☐	Human bocavirus 1 strain 3514-Dec2011 VP1 (VP1) and VP2 (VP2) genes, complete cds	790	790	99%	0.0	97%	KR014449.1
☐	Human bocavirus 1 strain 1521-Feb2010 VP1 (VP1) and VP2 (VP2) genes, complete cds	790	790	99%	0.0	97%	KR014439.1
☐	Human bocavirus 1 strain 1255-Feb2010 VP1 (VP1) and VP2 (VP2) genes, complete cds	790	790	99%	[illegible]	[illegible]	[illegible]
☐	[illegible]	[illegible]	[illegible]	[illegible]	[illegible]	[illegible]	[illegible]

Questions/comments

Sapovirus Std

Edit and Resubmit   Save Search Strategies   Formatting options   Download   How to read this page   Blast report description

Job title: Nucleotide Sequence (360 letters)

RID 0WEPBRCX01R (Expires on 12-11 17:07 pm)

Query ID lcl|Query_104081
Description None
Molecule type nucleic acid
Query Length 360

Database Name nr
Description Nucleotide collection (nt)
Program BLASTN 2.8.1+ Citation

Other reports: Search Summary [Taxonomy reports] [Distance tree of results] [MSA viewer]

Graphic Summary

Distribution of the top 100 Blast Hits on 100 subject sequences

Mouse over to see the title, click to show alignments

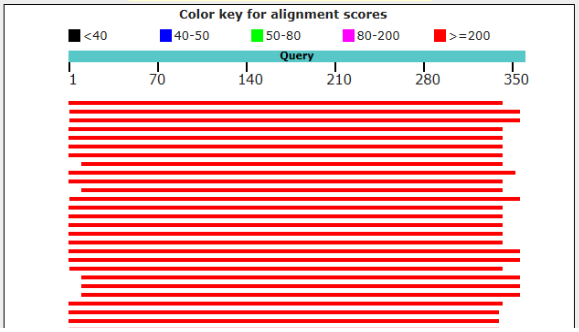

Questions/comments

Alignments   Download   GenBank   Graphics   Distance tree of results

	Description	Max score	Total score	Query cover	E value	Ident	Accession
☐	Sapovirus Hu/Pune/NIV075904/2007/India VP1 (VP1) gene, partial cds	448	448	95%	6e-122	91%	KU317433.1
☐	Sapovirus Mc2, complete genome	444	444	98%	7e-121	90%	AY237419.2
☐	Human calicivirus SLV/Bristol/98/UK gene for polyprotein and ORF2, genomic RNA	440	440	98%	3e-119	89%	AJ249939.1
☐	Sapovirus Hu/Pune/NIV076106/2007/India VP1 (VP1) gene, partial cds	439	439	95%	3e-119	90%	KU317436.1
☐	Sapovirus Hu/Pune/NIV117296/2011/India VP1 (VP1) gene, partial cds	434	434	95%	1e-117	90%	KU317449.1
☐	Sapovirus Hu/Pune/NIV1031131/2010/India VP1 (VP1) gene, partial cds	434	434	95%	1e-117	90%	KU317447.1
☐	Sapovirus Hu/Pune/NIV094581/2009/India VP1 (VP1) gene, partial cds	434	434	95%	1e-117	90%	KU317446.1
☐	Sapovirus Hu/S08219/2007/DNK capsid protein gene, partial cds	434	434	92%	1e-117	91%	GQ340721.1
☐	Sapovirus GII strain Hu/GII.1/Delhi/2014 capsid protein (VP1) gene, partial cds	430	430	97%	2e-116	89%	KY471140.1
☐	Sapovirus Hu/Pune/NIV0916007/2009/India VP1 (VP1) gene, partial cds	430	430	95%	2e-116	90%	KU317445.1
☐	Sapovirus Hu/CMH002/00/2000/THA capsid protein gene, partial cds	429	429	92%	5e-116	91%	EU363876.1
☐	Sapovirus GII strain Hu/GII.1/Novosibirsk/NS12-N4021/2012/RUS polyprotein gene, partial cds	426	426	98%	2e-115	89%	MF589697.1
☐	Sapovirus Hu/Pune/NIV071595/2007/India VP1 (VP1) gene, partial cds	425	425	95%	6e-115	90%	KU317435.1
☐	Sapovirus GII.1 gene for VP1 capsid protein, partial cds, strain: Sav GII/Hu/JP/2013/GII.1/OH13045	425	425	95%	6e-115	90%	LC081171.1
☐	Sapovirus Hu/GII.1/Aichi800-13/2014 ORF2 gene for capsid protein, partial cds	425	425	95%	6e-115	90%	LC086704.1
☐	Sapovirus GII NARA_0353_2014_JP VP1 gene for capsid protein, partial cds	425	425	95%	6e-115	90%	LC071912.1
☐	Sapovirus GII NARA_0215_2010_JP VP1 gene for capsid protein, partial cds	425	425	95%	6e-115	90%	LC071861.1
☐	Sapovirus GII.1 strain Hu/US/2015/GII.1/Nashville9346 ORF1 polyprotein and ORF2 protein genes, complete cds	424	424	98%	2e-114	88%	MG012445.1
☐	Sapovirus GII.1 strain Hu/US/2013/GII.1/Nashville9691-2 ORF1 polyprotein and ORF2 protein genes, partial cds	424	424	98%	2e-114	88%	MG012408.1
☐	Sapovirus Hu/Chiba/010774/2001/JP partial ORF1 gene for polyprotein	424	424	94%	[illegible]	[illegible]	[illegible]

Questions/comments

Aichivirus Std

BLAST Results

Edit and Resubmit Save Search Strategies ▷Formatting options ▷Download   How to read this page Blast report description

Job title: Nucleotide Sequence (630 letters)

**RID**	1EK5J8E1014 (Expires on 12-18 14:13 pm)		
**Query ID**	lcl|Query_196677	**Database Name**	nr
**Description**	None	**Description**	Nucleotide collection (nt)
**Molecule type**	nucleic acid	**Program**	BLASTN 2.8.1+ ▷Citation
**Query Length**	630		

Other reports: ▷Search Summary [Taxonomy reports] [Distance tree of results] [MSA viewer]

Graphic Summary

Distribution of the top 100 Blast Hits on 100 subject sequences

Mouse over to see the title, click to show alignments

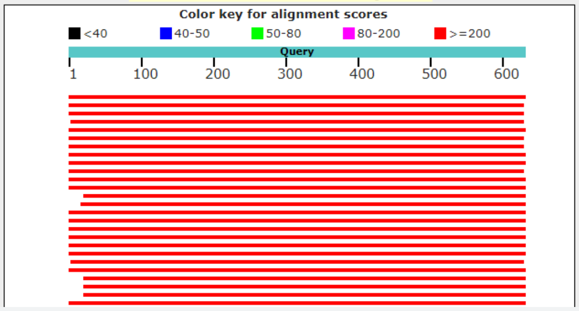

Questions/comments

https://blast.ncbi.nlm.nih.gov/Blast.cgi

Sequences producing significant alignments:

Select: All None Selected:0

Alignments Download GenBank Graphics Distance tree of results

Description	Max score	Total score	Query cover	E value	Ident	Accession
Aichi virus strain Pune/047811/India capsid protein gene, partial cds	1137	1137	100%	0.0	100%	HM363006.1
Aichi virus isolate B-302 polyprotein gene, partial cds	1043	1043	99%	0.0	97%	EU143276.2
Aichi virus isolate Goiania/GO/03/01/Brazil, complete genome	1039	1039	99%	0.0	97%	DQ028632.1
Aichi virus strain Pune/044963/India capsid protein gene, partial cds	1024	1024	99%	0.0	96%	HM363007.1
Aichivirus A isolate CMHA032-08 polyprotein gene, partial cds	1020	1020	100%	0.0	96%	KF414960.1
Aichi virus isolate B-101 polyprotein gene, partial cds	1016	1016	99%	0.0	96%	EU143274.2
Aichi virus isolate Chshc7, complete genome	1012	1012	99%	0.0	96%	FJ890523.1
Aichivirus A strain AUS/2008/Queensland FSS269 polyprotein gene, partial cds	1011	1011	100%	0.0	96%	MG253824.1
Aichivirus A strain AUS/2008/Queensland FSS266 polyprotein gene, partial cds	1011	1011	100%	0.0	96%	MG253823.1
Aichivirus A strain Qld-FSS698 polyprotein-like gene, partial sequence	1007	1007	99%	0.0	96%	MG237919.1
Aichivirus A strain AUS/2008/Queensland FSS271 polyprotein gene, partial cds	1006	1006	100%	0.0	95%	MG253825.1
Aichivirus A strain AUS/2008/Queensland FSS264 polyprotein gene, partial cds	1002	1002	100%	0.0	95%	MG253822.1
Aichi virus strain Pune/055326/India capsid protein gene, partial cds	1002	1002	96%	0.0	96%	HM363008.1
Aichivirus A strain AUS/2008/Queensland FSS699 polyprotein gene, partial cds	989	989	97%	0.0	96%	MG253826.1
Kobuvirus sp. strain 16806x8 polyprotein gene, partial cds	988	988	100%	0.0	95%	MF947442.1
Aichivirus A isolate CMHA317-08 polyprotein gene, partial cds	988	988	100%	0.0	95%	KF414961.1
Aichi virus strain D/VI2244/2004 polyprotein gene, complete cds	988	988	100%	0.0	95%	GQ927712.2
Aichi virus strain D/VI2321/2004 polyprotein gene, complete cds	988	988	100%	0.0	95%	GQ927706.2
Aichi virus strain D/VI2359/2004 polyprotein gene, complete cds	988	988	100%	0.0	95%	GQ927705.2
Aichi virus strain D/VI2169/2004 polyprotein gene, complete cds	988	988	100%	0 [illegible]	[illegible]	[illegible]
Aichi virus isolate B-171 polyprotein gene, partial cds	985	985	99%	0 [illegible]	[illegible]	[illegible]

Questions/comments
